# Combination antiretroviral therapy improves cognitive performance and functional connectivity in treatment-naïve HIV-infected individuals

**DOI:** 10.1007/s13365-017-0553-9

**Published:** 2017-08-08

**Authors:** Yuchuan Zhuang, Xing Qiu, Lu Wang, Qing Ma, Mark Mapstone, Amneris Luque, Miriam Weber, Madalina Tivarus, Eric Miller, Roberto C. Arduino, Jianhui Zhong, Giovanni Schifitto

**Affiliations:** 1 Department of Electrical and Computer Engineering, University of Rochester, Rochester, NY USA; 2 Department of Biostatistics and Computational Biology, School of Medicine and Dentistry, University of Rochester Medical Center, Rochester, NY USA; 3 Department of Pharmacy Practice, University at Buffalo, Buffalo, NY USA; 4 Department of Neurology, University of California, Irvine School of Medicine, Irvine, CA USA; 5 Department of Internal Medicine, University of Texas Southwestern Medical Center, Dallas, TX USA; 6 Department of Neurology, School of Medicine and Dentistry, University of Rochester Medical Center, Rochester, NY USA; 7 Department of Imaging Sciences, School of Medicine and Dentistry, University of Rochester Medical Center, 601 Elmwood Ave, Box 673, Rochester, NY 14642 USA; 8 Department of Psychiatry and Bio-behavioral Sciences, University of California, Los Angeles, CA USA; 9 Division of Infectious Diseases, Department of Internal Medicine, The University of Texas Health Science Center at Houston, Houston, TX USA

**Keywords:** HIV infection, Combination antiretroviral therapy, Functional magnetic resonance imaging, Diffusion tensor imaging, Cognitive function

## Abstract

Our study aimed to investigate the short-term effect of combination antiretroviral therapy (cART) on cognitive performance and functional and structural connectivity and their relationship to plasma levels of antiretroviral (ARV) drugs. Seventeen ARV treatment-naïve HIV-infected individuals (baseline mean CD4 cell count, 479 ± 48 cells/mm^3^) were age matched with 17 HIV-uninfected individuals. All subjects underwent a detailed neurocognitive and functional assessment and magnetic resonance imaging. HIV-infected subjects were scanned before starting cART and 12 weeks after initiation of treatment. Uninfected subjects were assessed once at baseline. Functional connectivity (FC) was assessed within the default mode network while structural connectivity was assessed by voxel-wise analysis using tract-based spatial statistics (TBSS) and probabilistic tractography within the DMN. Tenofovir and emtricitabine blood concentration were measured at week 12 of cART. Prior to cART, HIV-infected individuals had significantly lower cognitive performance than control subjects as measured by the total Z-score from the neuropsychological tests assessing six cognitive domains (*p* = 0.020). After 12 weeks of cART treatment, there remained only a weak cognitive difference between HIV-infected and HIV-uninfected subjects (*p* = 0.057). Mean FC was lower in HIV-infected individuals compared with those uninfected (*p* = 0.008), but FC differences became non-significant after treatment (*p* = 0.197). There were no differences in DTI metrics between HIV-infected and HIV-uninfected individuals using the TBSS approach and limited evidence of decreased structural connectivity within the DMN in HIV-infected individuals. Tenofovir and emtricitabine plasma concentrations did not correlate with either cognitive performance or imaging metrics. Conclusions: Twelve weeks of cART improves cognitive performance and functional connectivity in ARV treatment-naïve HIV-infected individuals with relatively preserved immune function. Longer periods of observation are necessary to assess whether this effect is maintained.

**Electronic supplementary material:**

The online version of this article (doi:10.1007/s13365-017-0553-9) contains supplementary material, which is available to authorized users.

## Introduction

HIV infection is associated with injury of the central nervous system (CNS) (Navia et al. 1986). The clinical manifestations of CNS injury vary from a mild decrease in cognitive performance without associated functional deficits to dementia (Antinori et al. 2007). Neuroimaging biomarkers provide an opportunity to investigate CNS injury especially at the time when clinical changes may be small or silent (Zhu et al. 2013). Most of the recent literature on HIV-associated cognitive impairment that has included neuroimaging is derived from patient with long established HIV infection and on stable combination antiretroviral treatment (cART) (Hua et al. 2013). It has been reassuring that the cognitive deficits of those on cART are often mild and the neuroimaging abnormalities are limited. However, we still have limited understanding of the causes for the persistence of cognitive impairment in HIV-infected individuals who have been virologically suppressed for years. One plausible explanation is that irreversible CNS damage occurs very early in HIV infection which, when limited, causes subtle cognitive changes measurable to some degree with extensive neuropsychological tests. In this case, effective cART would prevent progression of CNS injury but not reverse previous damage (Tozzi et al. 2005). Previous reports on cognitive improvement (not necessarily resolution) in patients with dementia after starting cART support this possibility (Chang et al. 1999). However, it is also possible that long-term treatment with cART may also induce neurotoxicity (Robertson et al. 2010). A bimodal response could be occurring where uncontrolled viral infection and associated inflammation would greatly benefit from cART resulting in cognitive improvement and decreased abnormalities in imaging metrics (Chang et al. 1999). To study the early time course progression of CNS injury in HIV infection, we focused on the default mode network (DMN) as the major region to investigate because it has been widely studied and reported to be affected by HIV infection (Becker et al. 2013; Herting et al. 2015; Ortega et al. 2015; Thomas et al. 2013). Here, we report our initial findings, assessing whether cognitive, functional, and structural brain connectivity were altered prior to the initiation of cART and whether there were measurable changes after 12 weeks of cART treatment.

## Materials and methods

All subjects, who consented to participate in the study, underwent a comprehensive clinical, laboratory (chemistry, hematology, and urine analysis), neurocognitive, and neuroimaging evaluation. HIV-infected individuals were assessed before and 12 weeks after starting cART, while HIV-uninfected controls were assessed only at baseline.

### Inclusion criteria

All participants were ≥18 years of age and antiretroviral (ARV) treatment-naïve prior to enrollment. They met the following laboratory parameters within 30 days of baseline evaluation: hemoglobin ≥9.0 g/dL, serum creatinine ≤2× ULN, AST (SGOT), ALT (SGPT), and alkaline phosphatase ≤2× upper limit of normal. Subjects were instructed to avoid smoking and use of caffeinated drinks for at least 2 h prior to the scheduled imaging section.

### Exclusion criteria

Participants with severe premorbid or comorbid psychiatric disorders were excluded. Subjects with mild or stable depression including those on stable antidepressant therapy were eligible for this study. Additional exclusion criteria were stroke, head trauma resulting in loss of consciousness >30 min, multiple sclerosis, brain infections (except for HIV-1), and any space-occupying brain lesions requiring acute or chronic therapy. Dementia, as established by HAND definition (Antinori et al. 2007), was exclusionary. Subjects meeting criteria for HIV-associated mild neurocognitive disorder (MND) or HIV-associated asymptomatic neurocognitive impairment (ANI) were eligible to participate. Active alcohol and drug abuse (urine toxicology was done at each visit) within 6 months of study entry and conditions such as claustrophobia or metallic implant that prevented MRI scanning were exclusionary.

### Neuropsychological tests

The neurocognitive evaluation was performed by trained staff and supervised by a neuropsychologist and included tests of executive function (Trailmaking Test Part B, Stroop Interference Task), speed of information processing (Symbol Digit Modalities Test and Stroop Color Naming), attention and working memory (CalCAP(CRT4) and WAIS-III Letter-Number Sequencing), learning (Rey Auditory Verbal Learning Test AVLT (trials 1–5), Rey Complex Figure Test Immediate Recall), memory (Rey Auditory Verbal Learning Test RAVLT Delayed Recall, Rey Complex Figure Test Delayed Recall), and motor (Grooved Pegboard, the left and right hands). An estimate of premorbid intellectual functioning ability was obtained via WRAT-4 Reading. The total composite Z-score was the primary cognitive outcome and was created from the linear combination of the Z-scores of the six cognitive domains measured (executive function, speed of information processing, attention and working memory, learning, memory, and motor). HAND diagnoses were determined for each participant according to the Frascati criteria (Antinori et al. 2007).

### MRI data acquisition

MRI was performed on a 3T Siemens MAGNETOM Trio MRI scanner equipped with a 32-channel head coil at the Rochester Center for Brain Imaging. A T1-weighted three-dimensional magnetization-prepared rapid acquisition gradient echo (MPRAGE, repetition time (TR)/inversion time (TI)/echo time (TE) = 2530/1100/3.44 ms, voxel size = 1.0 × 1.0 × 1.0 mm^3^, flip angle = 78°, bandwidth = 190 Hz/pixel) was acquired. DTI scans were acquired with the following parameters: 10 b = 0 s/mm^2^ images acquired increase signal to noise ratio; 60 diffusion weighting images which uniformly distributed with b = 1000 s/mm^2^; voxel size = 2 × 2 × 2 mm^3^; matrix size = 128 × 128. A double-echo gradient echo field map sequence was acquired with the same resolution as the DTI sequence and was used to correct for distortion caused by B0 inhomogeneity (Dietrich et al. 2008). The resting-state fMRI scans were acquired using a gradient echo-planar imaging (EPI) sequence (TR = 2000 ms, TE = 30 ms, flip angle = 90°, voxel size = 4 × 4 × 4 mm^3^; matrix size = 64 × 64, 30 axial slices, 150 time points). During the entire 5-min resting-state fMRI scanning, participants were instructed to keep their eyes open and avoid falling asleep.

### Resting-state fMRI processing

The first ten volumes were discarded for each subject to allow for stabilization of the magnetic field. Standard pre-processing steps were performed using Data Processing Assistant for Resting-State fMRI (DPARSF) (Chao-Gan and Yu-Feng 2010) and included the following: slice timing correction, motion correction, normalization, and spatial smoothing with Gaussian kernel (full width at half maximum (FWHM) = 4 mm), linear trend removal, and band-pass filter (0.01 Hz to 0.08 Hz) to reduce the low-frequency drift and exclude physiological noise. We used maximum head motion displacement larger than 2 mm and head rotation greater than 2° as exclusion criteria. No subjects were excluded in this cohort. Each individual’s fMRI images were normalized to Montreal Neurological Institute (MNI) standard space. To minimize head motion effect and non-neuronal noise, nuisance covariates including six head motion parameters, global mean signal, white matter time series, and CSF time series were regressed out. After pre-processing, group independent component analysis (ICA) was performed to identify the default mode network (DMN) using Group ICA part of fMRI Toolbox (GIFT, http://mialab.mrn.org/software/gift/index.html). All subjects’ resting-state fMRI data were included in a single group ICA analysis. Data were reduced through principal component analysis (PCA) (Calhoun et al. 2001; Erhardt et al. 2011) in two stages: one at the single subject level and a second one at the group level. Following data reduction, the multi-subject ICA was performed to identify the independent component using Infomax algorithm (Bell and Sejnowski 1995). GICA-based back-reconstruction methods were used to reconstruct single-subject components. This resulted in 20 independent component (IC) spatial maps for each subject.

The single DMN component was firstly identified by visual inspection. The one-sample *t* test was used to test the significance of the selected DMN component that resulted in a t-map. Only the posterior DMN component which includes the posterior cingulate cortex (PCC) and the inferior parietal cortex (IPC) were included after using *p* < 0.0001 with FDR correction.

The average time series of all the voxels in the DMN was calculated and correlated with each voxel’s own time series within DMN, to calculate within-network functional connectivity (Supekar et al. 2010). Fisher’s r-to-z transformation was applied to the functional connectivity for each individual.

### Diffusion tensor imaging processing

The first ten b = 0 images and 60 diffusion-weighted images were firstly corrected for motion and eddy-current-induced distortion, using eddy correct tool in FSL (Smith et al. 2004; Woolrich et al. 2009; Jenkinson et al. 2012). The ten b = 0 images were averaged as a b0 image, which was then used to register DTI data to T1 structural image using boundary-based registration (BBR) (Greve and Fischl 2009) in FSL. Brain extraction was performed on the averaged b0 image using BET (Smith 2002) in FSL. The averaged b0 image and 60 DWIs were used to calculate the tensor-derived scalar metrics including fractional anisotropy (FA), mean diffusivity (MD), radial diffusivity (RD), and axial diffusivity (AD) using DTIFIT in FSL. A brain mask was applied on each tensor-derived scalar metric to create brain-extracted maps.

Voxel-wise analysis was performed using tract-based spatial statistics (TBSS) (Smith et al. 2006), part of FSL. Group comparisons were performed using the Randomize toolbox (Nichols and Holmes 2002; Winkler et al. 2014) in FSL, and the threshold-free cluster enhancement (TFCE) approach (Smith and Nichols 2009) was used for multiple comparison correction. We also performed probabilistic tractography within DMN using FSL. A DMN mask was manually selected from Group ICA analysis using resting-state fMRI: PCC served as seed region, and medial prefrontal cortex (mPFC), left- and right-inferior parietal cortex (IPC) served as target regions, respectively. The structural connectivity (SC) was calculated in two ways, the total number of voxel within the tracts, and the mean FA within the tracts. (See Supplemental material for details).

### Drug concentration measurement and pharmacokinetic analysis

Plasma tenofovir (TFV) and emtricitabine (FTC) were measured using a newly developed and validated liquid chromatography with tandem mass spectrometry (LC-MS/MS) method (Delahunty et al. 2009). TFV and FTC were chromatographically separated on the LC system, detected at the tandem MS system and calibrated within a linear range of 10–1000 ng/mL. The lower limit of quantitation for TFV and FTC was determined to be 10.0 ng/mL, and assay precision was <10%. The study participants were taking ARV regimens that included tenofovir disoproxil fumarate (TDF) 300 mg daily and FTC 200 mg daily for at least 12 weeks. In order to estimate TFV and FTC exposure, population models were developed using the data from the AIDS Clinical Trials Group study A5202 (Beal 2001; Sax et al. 2009; Valade et al. 2014). TFV and FTC pharmacokinetics at the steady state was best described by a two-compartment linear mammillary model with first-order absorption with inter-individual variability on clearance. The exposure was indicated by the area under the plasma drug concentration-time curve (AUC, mg/L*h).

### Statistical analysis

Data are presented as mean ± SE throughout this manuscript. Comparisons between two independent groups were subjected to Wilcoxon rank-sum test. For comparing measurements collected from the same subject at two time points, Wilcoxon signed-rank test was used instead. Spearman correlation test was used for testing the dependence of two continuous variables. A *p* value <0.05 was considered statistically significant.

All analyses were performed in R 3.2.0 (R Foundation for Statistical Computing, Vienna, Austria) and in SAS 9.3 (SAS Institute, Cary NC).

## Results

Seventeen ARV treatment-naïve HIV-infected individuals were age matched with 17 HIV-uninfected individuals. HIV-infected individuals were scanned before starting cART, and, on average, 12 weeks after the initiation of ARV treatment. Demographics and baseline clinical characteristics are shown in Table 1. The groups were well balanced for age and education. The uninfected group was more likely to be female and White. At baseline, ten HIV-infected individuals had normal cognitive performance, six had ANI, and one had MND. Applying HAND classification to HIV-uninfected individuals, there was a similar proportion of cognitive impairment. However, when using the composite Z-score of all cognitive tests, HIV-infected individuals performed worse than HIV-uninfected individuals (Table 2). The mean CD4 cell count and HIV RNA levels at baseline were 479 ± 48 cells/mm^3^ and 5.08 ± 4.64 log_10_ copies/mL, respectively. The relatively high CD4 cell count in the HIV-infected individuals at baseline likely reflects a recent HIV infection; however, timing from exposure to HIV to diagnosis was not available. After 12 weeks, mean CD4 cell count and HIV RNA levels were 636 ± 55 cells/mm^3^ and 899 ± 861 copies/mL, respectively.

**Table 1:** Demographics and baseline clinical variables

	HIV infected (*n* = 17)	HIV uninfected (*n* = 17)	*p* value
Age ((years) mean ± SE)	32.6 ± 2.9	32.4 ± 3.0	0.956
Gender (M:F)	17:0	7:10	<0.001
Ethnicity (White:Black:other)	8:9:0	12:3:2	0.042
Education			
≤12 years	4	3	1
>12 years	13	14	
HIV duration by patient report at baseline ((months) median (range))	1 (1, 120)	NA	–
Baseline CD4 cell count ((cells/mm^3^) mean ± SE)	479.2 ± 48.5	NA	–
Baseline HIV RNA levels ((log_10_ unit) median (range))	4.7 (1.7, 5.8)	NA	–
HAND classification at baseline			
Normal	10	11	1
ANI	6	5	
MND	1	1	

*HAND* HIV-Associated Neurocognitive Disorder, *ANI* Asymptomatic Neurocognitive Impairment, *MND* Mild Neurocognitive Disorder

**Table 2:** Cognitive performance by composite Z-score cognitive domain at baseline in HIV-uninfected individuals and in HIV-infected individuals before and after 12 weeks on cART

Composite Z-score	HIV− baseline (mean ± SE)	HIV+ baseline (mean ± SE)	HIV+ 12 weeks (mean ± SE)	HIV− vs. baseline HIV+ (*p* value)^a^	HIV+ 12 weeks vs. HIV− (*p* value)^a^	HIV+ baseline vs. 12 weeks (*p* value)^b^
Executive function	0.30 ± 0.28	−0.24 ± 0.23	−0.06 ± 0.21	0.125	0.379	0.332
Speed of information processing	0.33 ± 0.21	−0.13 ± 0.25	−0.20 ± 0.26	0.179	0.158	1.000
Attention	0.21 ± 0.22	−0.23 ± 0.27	0.02 ± 0.23	0.343	0.617	0.309
Learning	0.09 ± 0.22	−0.13 ± 0.22	0.04 ± 0.29	0.502	0.667	0.404
Memory	0.00 ± 0.22	−0.12 ± 0.24	0.11 ± 0.27	0.730	0.654	0.094
Motor	0.53 ± 0.22	−0.29 ± 0.26	−0.24 ± 0.21	*0.023*	*0.025*	0.624
Total composite Z-score	1.46 ± 0.86	−1.14 ± 1.07	−0.32 ± 0.9	*0.020*	0.057	0.174

^a^Wilcoxon rank-sum test

^b^Wilcoxon signed-rank test

*p*-value < 0.05 were in italics

Antiretroviral regimens prescribed included Complera (rilpivirine, FTC, TDF, *n* = 2), Stribild (elvitegravir, cobicistat, FTC, TDF, *n* = 8), Atripla (efavirenz, FTC, TDF, *n* = 3), Truvada (FTC/TDF) plus Tivicay (dolutegravir, *n* = 1), Truvada (FTC/TDF) plus Isentress (raltegravir, *n* = 1), and Triumeq (dolutegravir, abacavir, lamivudine, *n* = 1).

The results in Table 2 show that the mild cognitive deficits present at baseline in HIV-infected compared with HIV-uninfected individuals, tended to improve in most cognitive domains, with exception of motor function, after 12 weeks of cART.

**Fig. 1:**
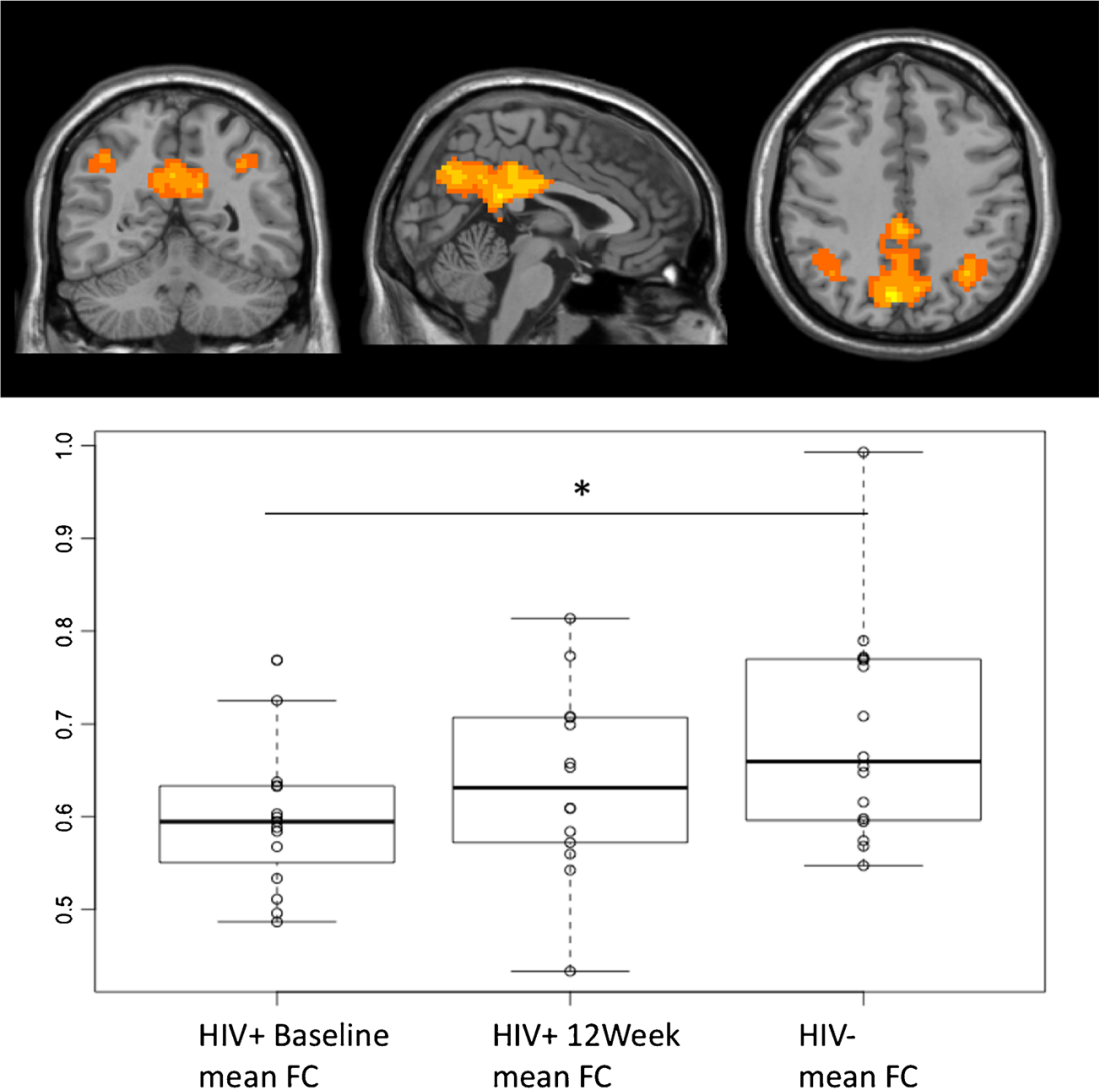
Functional connectivity (*FC*) within default mode network (DMN). Mean FC for HIV infected before treatment was 0.588 and increased to 0.637 after 12 weeks of cART, while the mean FC for HIV-uninfected group was 0.689; *p = 0.008

Functional connectivity (FC) was evaluated within the DMN. For these analyses, 16 HIV-infected and 16 HIV-uninfected individuals were available at baseline and 14 HIV-infected individuals at 12 weeks. Mean FC at baseline was 0.588 in HIV-infected and 0.689 in those uninfected (*p* value = 0.008). After 12 weeks of cART, mean FC increased to 0.637, and when compared with HIV-uninfected individuals, the difference was not significant (*p* value = 0.197). Figure 1 shows the DMN in this population as determined using ICA and the mean FC in the DMN for each group of subjects and visits.

**Fig. 2:**
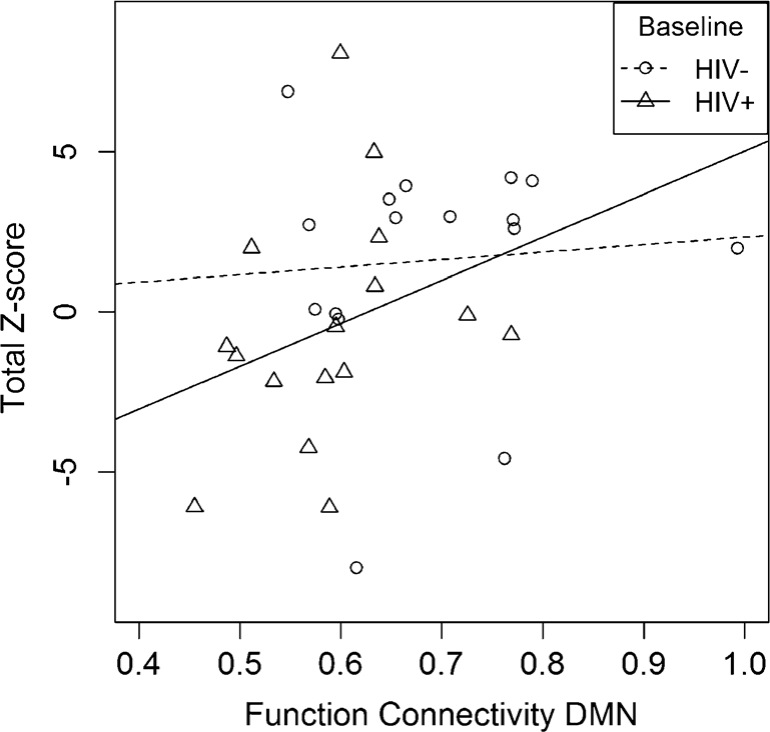
Functional connectivity correlation with cognitive performance. FC was significantly correlated with total composite Z-score for HIV-infected individuals at baseline (*ρ* = 0.50, *p* value = 0.046) but not with HIV-uninfected individuals

**Fig. 3:**
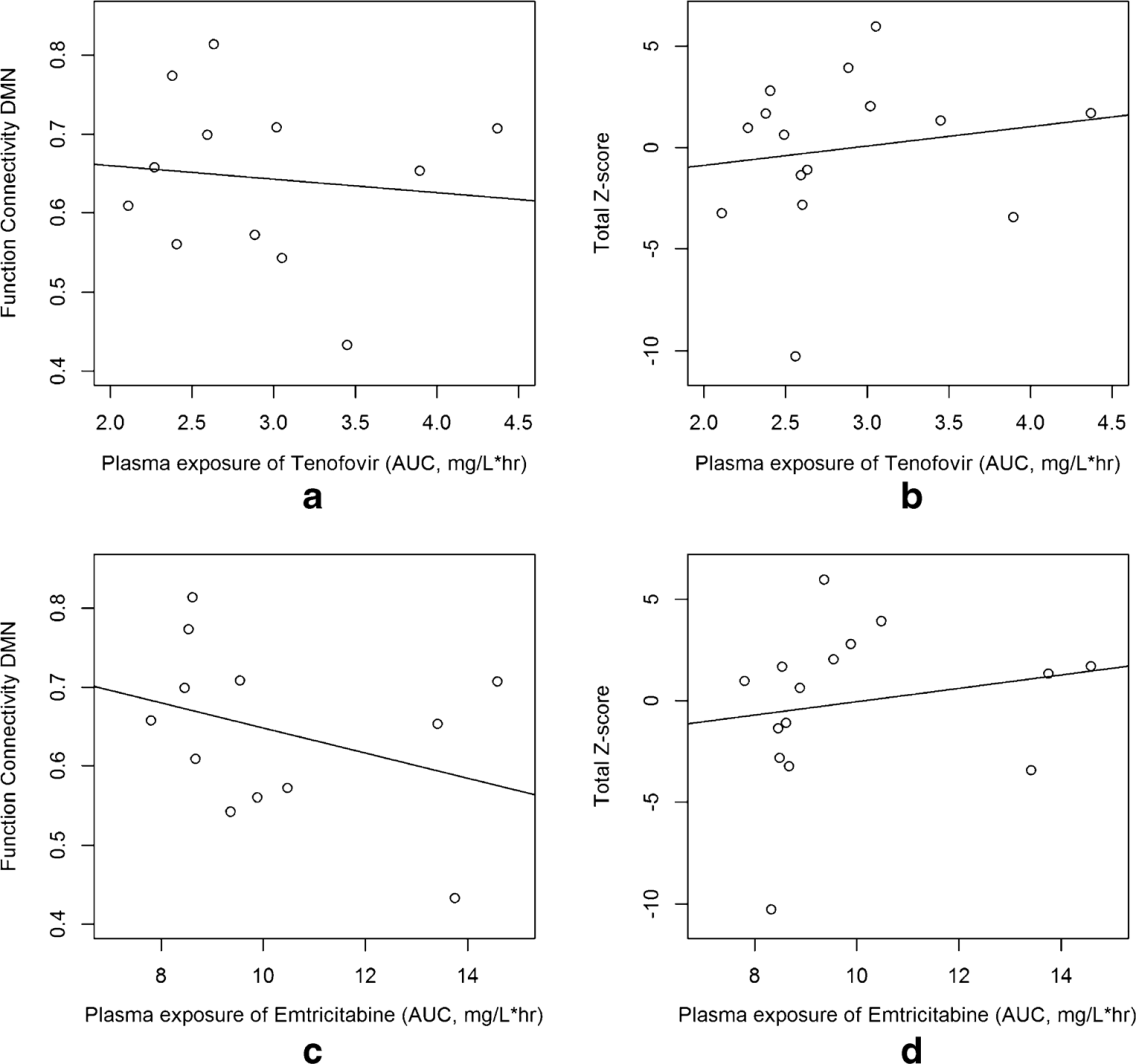
Correlations between plasma exposure of TFV, FTC, and FC and cognitive performance

We further examined the relationship between FC and cognitive performance. FC was significantly correlated with total Z-score in HIV-infected individuals at baseline (Spearman correlation 0.50, *p* value = 0.046) (see Fig. 2) but was not significantly correlated with either HIV-infected after 12 weeks of cART or HIV uninfected. Other variables such as CD4 cell count, HIV RNA levels, and the duration of HIV infection were not significantly correlated with FC (Fig. 3).

We also assessed whether the FC and total Z-score correlated with plasma exposure of TFV and FTC. For both TFV and FTC, there was no significant correlation between FC and plasma exposure (*p* value = 0.73 and 0.26, respectively) and Z-score and plasma exposure of these ARVs (*p* value = 0.58 and 0.51, respectively).

White matter microstructure abnormalities were assessed via diffusion tensor imaging and specifically via TBSS and probabilistic tractography within the DMN. No significant differences were found in TBSS analyses between HIV-infected and HIV-uninfected individuals nor in HIV-infected individuals before and after 12 weeks of cART. However, the results from the probabilistic tractography were inconclusive. While the total number of voxels from PCC to left-IPC was significantly greater in HIV-uninfected compared with HIV-infected both at baseline and 12 weeks of treatment, *p* < 0.01 and *p* < 0.05, respectively, the mean FA within the tract was not significant. In addition, we did not find significant differences in the other two pairs of tracts, in either number of voxels or mean FA. Lastly, there was no significant correlation between SC and FC.

## Discussion

There is still ongoing debate on whether long-term use of cART may have deleterious effect on brain structure and function (Robertson et al. 2010; Cysique et al. 2009; Smurzynski et al. 2011; Marra et al. 2009). Neuroimaging has been used as a non-invasive tool to assess CNS injury in HIV-infected individuals. A few studies have assessed the impact of HIV infection and cART on cognitive performance and imaging metrics using fMRI or diffusion tensor imaging in ARV treatment-naïve individuals in a cross-sectional design (Ortega et al. 2015; Chang et al. 2008; Ragin et al. 2015). Our study adds to this literature by evaluating a longitudinal design and ARV regimens that reflects current standard of practice in the USA. We found significant differences in cognitive performance, as measured by the total composite Z-score created from combination of all neuropsychological tests used, and functional connectivity (FC) within DMN in ARV treatment-naïve HIV-infected compared with HIV-uninfected individuals that improved after 12 weeks of cART. Of note, neither baseline HIV RNA levels nor CD4 cell count correlated with either FC or cognitive performance.

A recent cross-sectional study by Ortega et al. (2015) analyzed cognitive performance and FC in three groups of individuals: HIV-uninfected, HIV-infected on cART, and HIV-infected not on cART. The group not on cART was a mix of never on cART or currently not taking cART. The investigators found that HIV-infected individuals had lower FC than HIV-uninfected individuals in areas that included DMN, and this was even lower in those not on cART. Our results confirm these findings and provide longitudinal support that ARV treatment, at least in the short run, tends to normalize the FC. Importantly, we also show that cognitive performance correlated with FC before ARV treatment and that after treatment, improvement in cognitive performance rendered this correlation non-significant, similar to the observation in HIV-uninfected individuals.

While several studies have found some difference in FC, not necessarily in the same networks, between HIV-infected and HIV-uninfected individuals (Ortega et al. 2015; Thomas et al. 2013; Ipser et al. 2015; Wang et al. 2011), others have not (Janssen et al. 2016). However, these studies represent heterogeneous populations and analysis methods and thus are difficult to compare. Another study, for example, using a fMRI activation task, found that HIV-infected individuals on cART require greater brain activation than HIV-infected not on cART or HIV-uninfected, thus pointing to a possible deleterious effect of cART (Chang et al. 2008).

We and others have shown that HIV infection is associated with microstructural white matter abnormalities (Zhu et al. 2013; Ragin et al. 2005; Pfefferbaum et al. 2007; Pomara et al. 2001). These studies have included HIV-infected individuals with different degrees of immune suppression and comorbidities. The current study offers the opportunity to assess the effect of HIV viremia and cART on white matter in a longitudinal design in ARV treatment-naïve subjects with relative preserved immune function, comparing them very well with age-matched HIV-uninfected controls. We found no significant white matter microstructural differences (using TBSS) comparing HIV-infected with HIV-uninfected and HIV-infected before and after starting cART. These results differ from a recent cross-sectional study in patients recently infected, which found abnormalities in the corpus callosum (Ragin et al. 2015). One possible explanation is that the patients in our study had a relatively well-preserved immune function and both HIV-infected and HIV-uninfected individuals were tightly age matched. Group differences in gender, as observed in our study, are less likely to affect DTI metrics while age has a large impact (Wu et al. 2011). Given the small sample size, variability in immune function and age may have a large effect on results.

In order to more directly compare functional and structural connectivity, we assessed structural connectivity within the DMN using probabilistic tractography. The results of these analyses were inconclusive as we found decreased connectivity, when measured by number of voxel, in HIV-infected individuals only in the tract connecting PCC to left IPC. However, mean FA within this tract, the PCC to right IPC, and PCC to medial prefrontal cingulate cortex did not differ between HIV-infected and HIV-uninfected individuals.

We did not find a significant correlation between the plasma exposures of TFV and FTC with both cognitive performance and FC. These results do not exclude potential long-term effect as suggested by a recent small study (Ma et al. 2015) where higher plasma concentrations of TFV and FTC were found in patients with declining cognitive performance. The distribution of these agents into the brain, particularly TFV, is limited due to its anionic charge at physiological pH making it difficult to cross the blood–brain barrier. In fact, low TFV concentrations and accordingly high HIV RNA levels in cerebral spinal fluid (CSF) were reported in previous studies suggesting the CNS as a sanctuary for HIV viral replication and viral escape (Best et al. 2012; Calcagno et al. 2011).

Limitations in this study include small sample and short duration of the longitudinal follow-up. In addition, gender and ethnicity was significantly different in the two cohorts. However, these factors should have not significantly affected the neuroimaging results (Joel et al. 2015). Also, we cannot exclude that cognitive performance improved in the HIV-infected individuals secondary to a practice effect. The study design did not include a 12-week evaluation in the HIV-uninfected group as we did not expect cognition to change within that period. However, the change observed in FC in HIV-infected individuals is unlikely to be explained by practice effect.

In summary, our results suggest that in fairly young ARV treatment-naïve individuals with relatively preserved immune function, there are signs of decreased functional connectivity that correlates with decreased cognitive performance. There was inconclusive evidence of white matter microstructural abnormalities suggesting that the process may be reversible if HIV infection is treated early. In this regard, the improvement in cognitive performance and FC after 12 weeks of cART is encouraging and would favor the current approach of treating HIV infection as early as possible. However, a longer longitudinal follow-up and larger study is necessary to assess whether the improvement observed is sustainable overtime or whether the potential neurotoxicity of cART will manifest.

## Electronic supplementary material

ESM 1 (DOCX 12098 kb)
