# Supplementary material for: Combination antiretroviral therapy improves cognitive performance and functional connectivity in treatment-naïve HIV-infected individuals
Source: J Neurovirol. 2017 Aug 8;23(5):704–12. doi: 10.1007/s13365-017-0553-9 (PMC5655604; doi:10.1007/s13365-017-0553-9)
Supplement: Supplementary file 1 — (DOCX 12098 kb) [file 13365_2017_553_MOESM1_ESM.docx]

Method

The DMN mask for probabilistic tractography was extracted from the group ICA using one-sample t-test, p<0.001 with FDR correction. The four nodes in DMN were manually chosen as ROIs: posterior cingulate cortex (PCC), medial prefrontal cingulate cortex (mPFC), left and right inferior parietal cortex (l-IPC, r-IPC). The ROIs were registered to individual’s native DTI space using FLIRT with BBR in FSL(Greve and Fischl, 2009). The ROIs were binarized and expanded by 3 voxels at boundary, using mean dilation to include the neighboring white matter voxels, using fslmaths function in FSL. PCC was set as seed region, and the other three were set as target regions respectively(Khalsa *et al*, 2014).

We used probabilistic tractogrpahy(Behrens *et al*, 2007; Behrens *et al*, 2003) to quantify structural connectivity with FMRIB's Diffusion Toolbox (FDT, <https://fsl.fmrib.ox.ac.uk/fsl/fslwiki/FDT)>. Before running probabilistic tractography, Bedpostx in FSL was performed to estimate diffusion parameters at each voxel using Markov Chain Monte Carlo sampling. The probabilistic tractography was calculated using probtrackx in FSL. In each voxel within PCC mask, 1000 samples were performed with 2000 steps per sample, curvature threshold of 0.2, and a step length of 0.5. The mPFC, l-IPC, and r-IPC were used as waypoint masks respectively. Only tracts that pass through these target regions were included. This resulted in a connectivity distribution map, each voxel in this map were given by the number of tracts passing through this voxel, that reached to target mask from the seed mask. We normalized the connectivity distribution map by waytotal number, which is given by the total number of tracts generated from seed mask that are not being rejected by the waypoint mask criteria. We threshold the normalized connectivity map to 1%, 5%, and 10% to find the most likely location of the tracts. All voxels that passed thresholds of 1%, 5%, and 10% of the normalized connectivity distribution map reflect the most representative tracts between two regions. The number of voxels, as well as the mean FA value within the tracts were used to quantify the strength of the structural connectivity.

Statistical analysis

We compared the three pairs of structural connectivity within DMN between PCC-mPFC, PCC-l-IPC, and PCC-r-IPC using two-sample t-test. For each pair of tracts, the total number of voxels and the mean FA within each tract were compared. Pearson’s correlation test was performed to test the correlation between the structural connectivity and the FC value within DMN. A p-value < 0.05 was considered statistically significant.


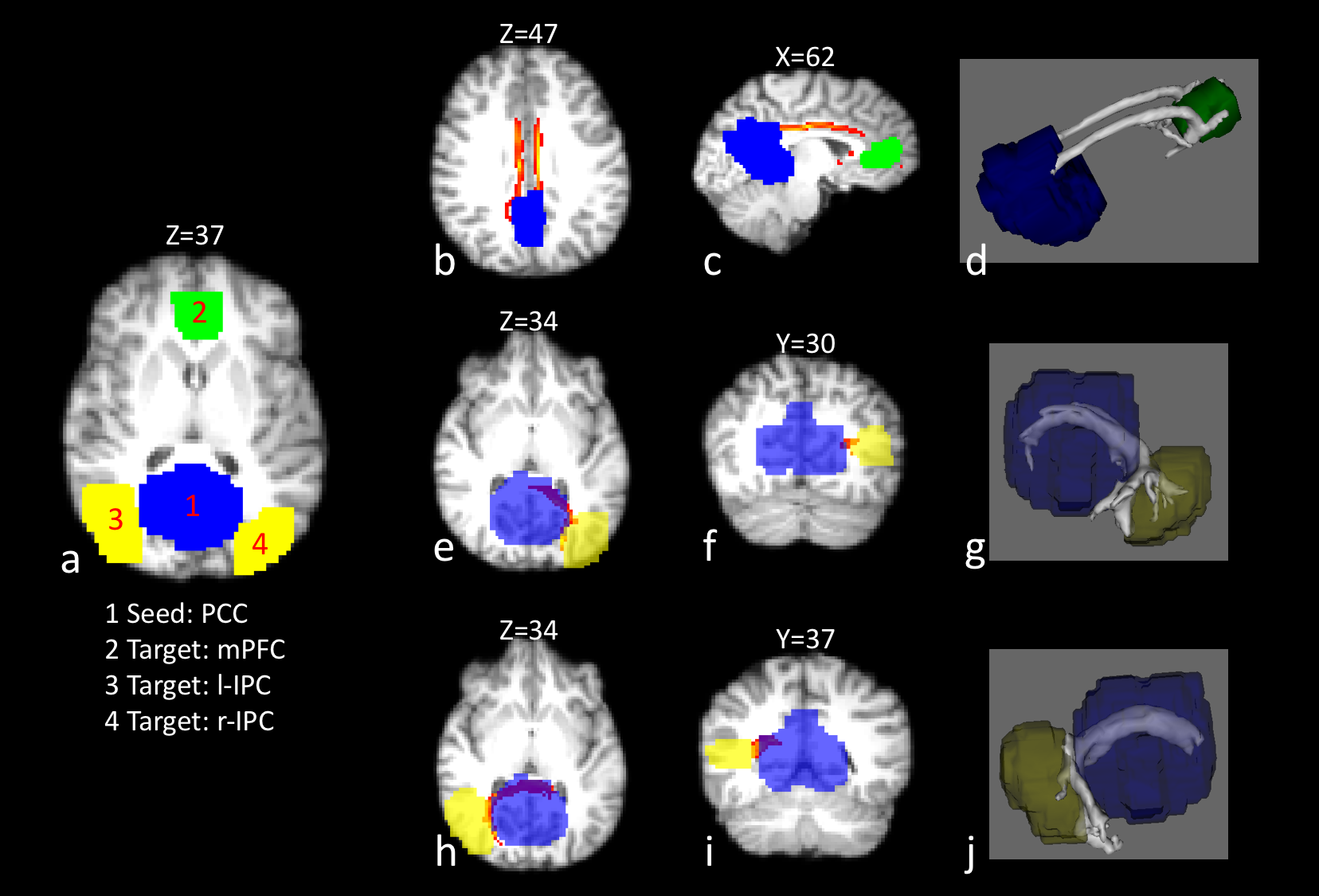


Figure S1. Examples of structural connectivity calculated using probabilistic tractography. a) the four nodes in DMN after registering to individual native DTI space, which served as seed and target regions for probabilistic tractogrpahy. The background image is the T1-weighted image registered to individual native DTI space. b) and c) show tracts (red-yellow) connecting PCC (blue) and mPFC (green). e) and f) show tracts (red-yellow) connecting PCC (blue) and l-IPC (yellow). h) and i) show tracts (red-yellow) connecting PCC (blue) and r-IPC (yellow). d), g), and j) show the 3D reconstructed tracts (grey) and nodes for PCC-mPFC, PCC-l-IPC, and PCC-r-IPC respectively.

Result

Only PCC-l-IPC tract showed a significant difference between HIV-infected and healthy control subjects when using total number of voxels that passed 1% threshold. The healthy control group showed significantly higher structural connectivity when compared with HIV-infected (compared to baseline and 12 week, p-value = 0.009 and 0.02 respectively). There were no significant differences in the other two pairs of tracts, for either number of voxels or mean FA values. We did not find any significant correlation between structural connectivity and FC.


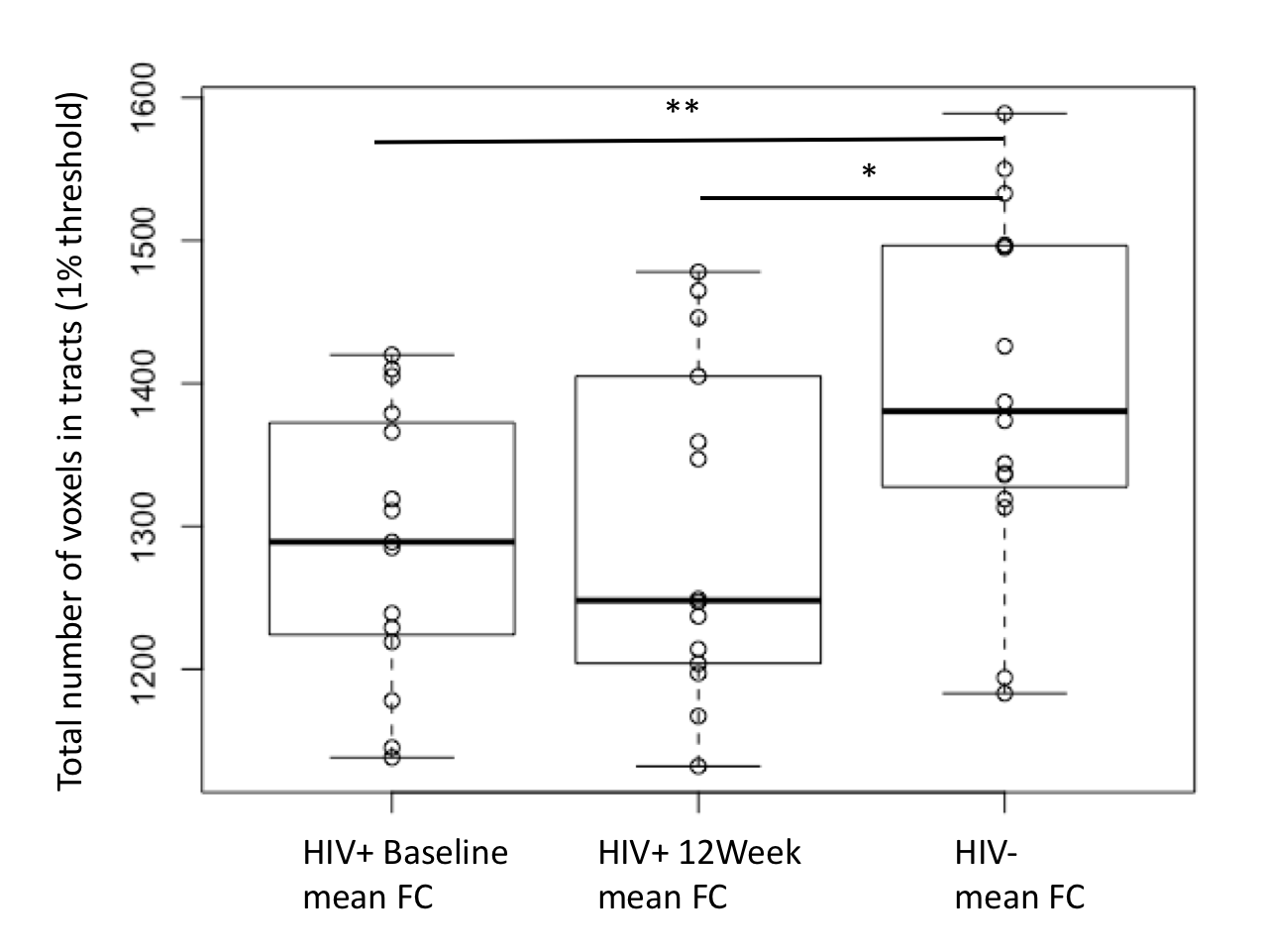


Figure S2. Structural connectivity expressed in total number of voxels in the tract between PCC and l-LPC in HIV-uninfected group is significantly stronger than in HIV-infected before treatment (**p<0.01), and after 12 weeks of treatment (*p<0.05).

Reference

Behrens TE, Berg HJ, Jbabdi S, Rushworth MF, Woolrich MW (2007). Probabilistic diffusion tractography with multiple fibre orientations: What can we gain? Neuroimage 34: 144-55.

Behrens TE, Woolrich MW, Jenkinson M, Johansen-Berg H, Nunes RG, Clare S, Matthews PM, Brady JM, Smith SM (2003). Characterization and propagation of uncertainty in diffusion-weighted MR imaging. Magn Reson Med 50: 1077-88.

Greve DN, Fischl B (2009). Accurate and robust brain image alignment using boundary-based registration. Neuroimage 48: 63-72.

Khalsa S, Mayhew SD, Chechlacz M, Bagary M, Bagshaw AP (2014). The structural and functional connectivity of the posterior cingulate cortex: comparison between deterministic and probabilistic tractography for the investigation of structure-function relationships. Neuroimage 102 Pt 1: 118-27.
